# Giant Cell Tumour of Proximal Phalanx of Ring Finger: Case Report and Review of Literature

**DOI:** 10.7759/cureus.835

**Published:** 2016-10-18

**Authors:** Rishit Soni, Chirag Kapoor, Malkesh Shah, Amit Patel, Paresh Golwala

**Affiliations:** 1 Orthopaedics, Sumandeep Vidyapeeth, Vadodara, Gujarat

**Keywords:** giant cell tumour, curettage, phalanx, bone graft

## Abstract

Giant cell tumour (GCT) of bone arising from a phalanx of a finger is extremely rare. Only two percent of all reported GCTs are found in the hand, which show a higher rate of recurrence as compared to those occurring at a more proximal location. Here we report a rare case of giant cell tumour of proximal phalanx of the ring finger in a 20-year-old male, which was treated with extended curettage and bone grafting. After two years of follow-up, the patient was asymptomatic with complete functional recovery and no signs of recurrence.

## Introduction

Giant cell tumour is an uncommon benign osseous tumour usually seen at the epiphysis of a long bone after skeletal maturity. It is defined as a benign but locally aggressive neoplasm [1]. Only two percent of all reported GCTs are found in the hand, with phalangeal bones being a very rare primary site of involvement. In reported cases, GCTs at a phalanx have shown quite a different behaviour with higher rates of recurrence as compared to those at more common locations like the distal femur. Local recurrence following simple curettage and bone grafting has been reported to be as high as 90% [2]. Herein we report a case of GCT of the proximal phalanx of the ring finger in a young male patient treated with extended curettage and bone grafting.

## Case presentation

A 20-year-old male patient presented with pain and swelling of the left ring finger base since five months without any history of trauma or constitutional symptoms. On examination, a fusiform swelling in the proximal phalanx of the left ring finger was noted, which was tender, firm in consistency, and had normal overlying skin without any scar or adherence to the underlying tissue. The adjacent metacarpophalangeal (MCP) and proximal interphalangeal (PIP) joints had normal ranges of movements.

**Figure 1:**
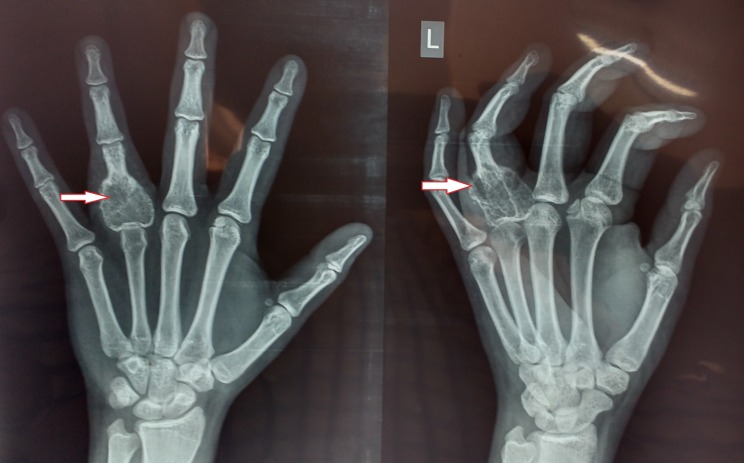
Pre-op radiograph (anteroposterior and oblique views) Shows lytic lesion at the base of the proximal phalanx of the ring finger

Radiographs showed an expansile lytic lesion involving the base and proximal half of the proximal phalanx shaft with a thin cortical rim (Figure 1). There were no signs of periosteal reaction with intact articular margins. The chest radiograph was normal and the laboratory investigations were within normal limits.

**Figure 2:**
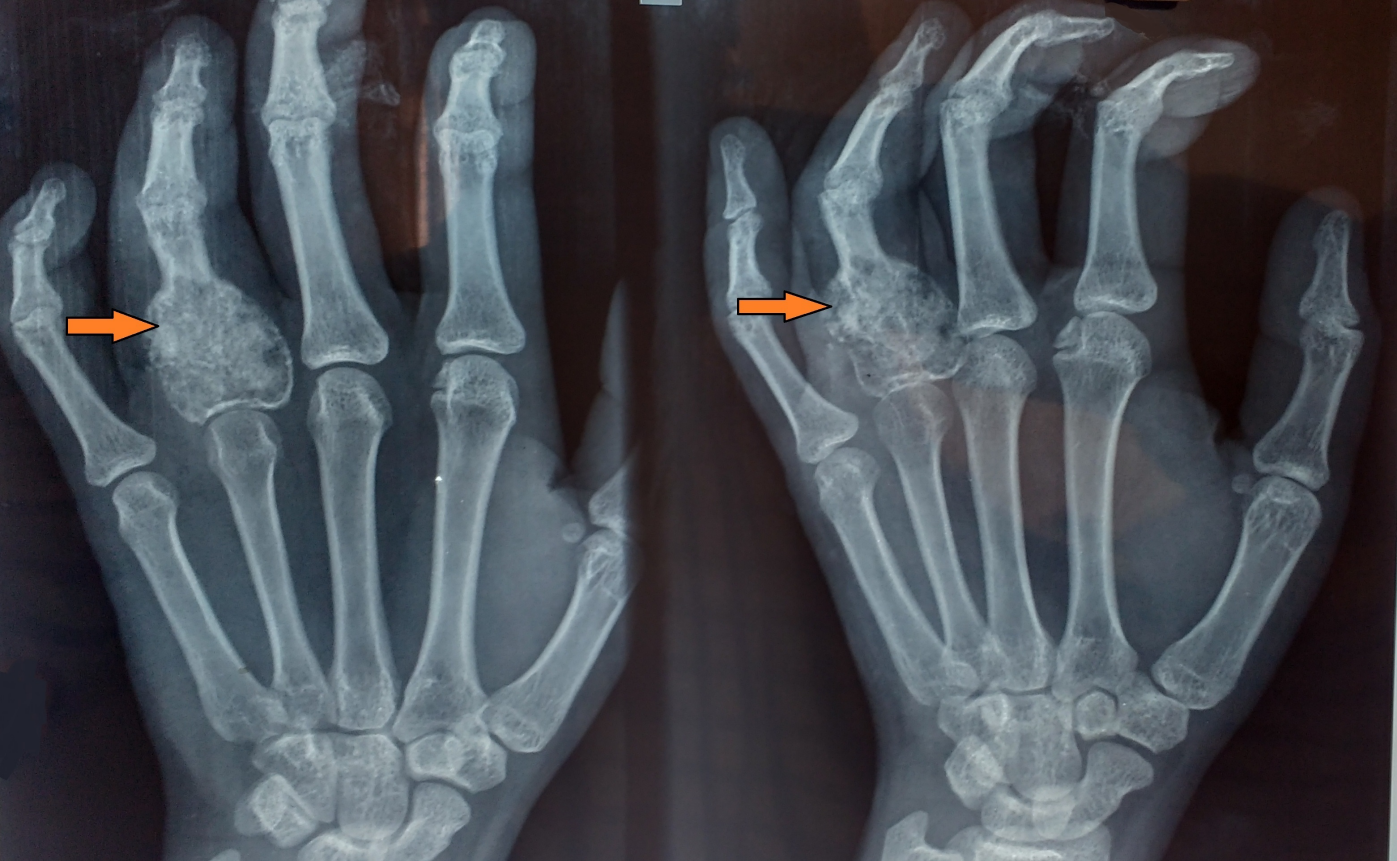
Post-op radiograph Shows curettage of the lesion and the bone grafting done

**Figure 3:**
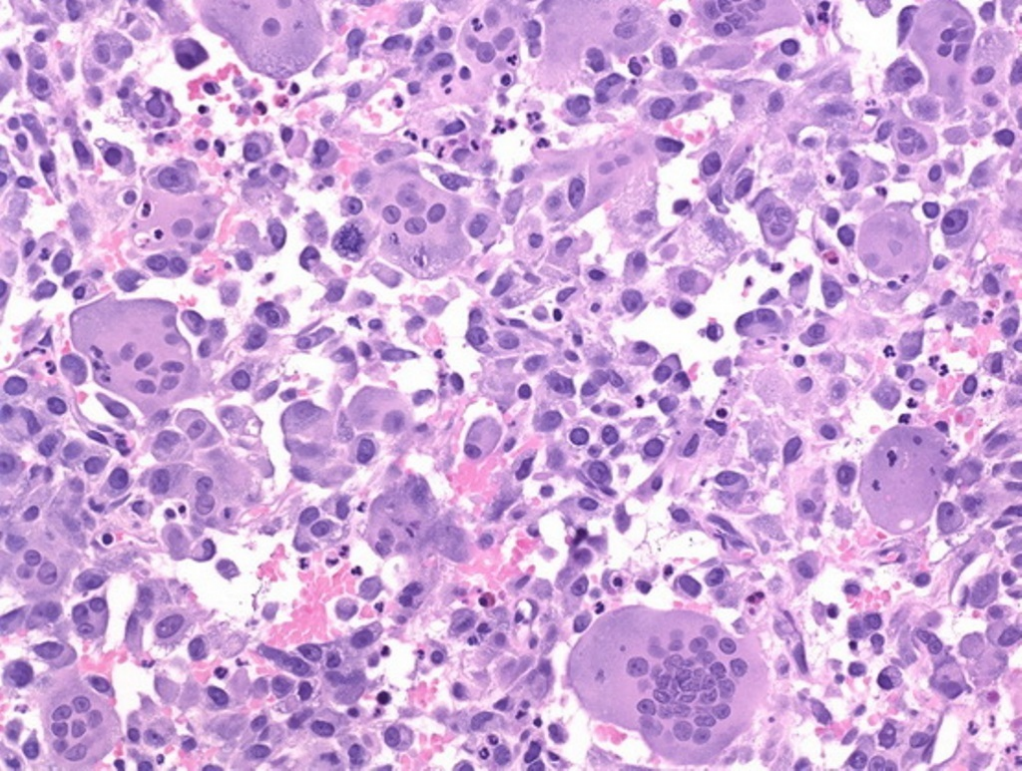
Histopathology slide

The differential diagnosis at this stage was giant cell tumour, enchondroma, and aneurysmal bone cyst. After giving a written informed consent, the patient underwent extended curettage of the lesion with chemical cauterization by phenol, and the void was filled with autologous cancellous bone graft taken from the iliac crest (Figure 2). A biopsy of bone tissue was sent for histopathological examination, which showed proliferation of osteoclast-type giant cells, uniformly distributed with mononucleated polygonal cells showing brisk mitotic activity at focal areas (Figure 3). This confirmed the diagnosis as giant cell tumour.

**Figure 4:**
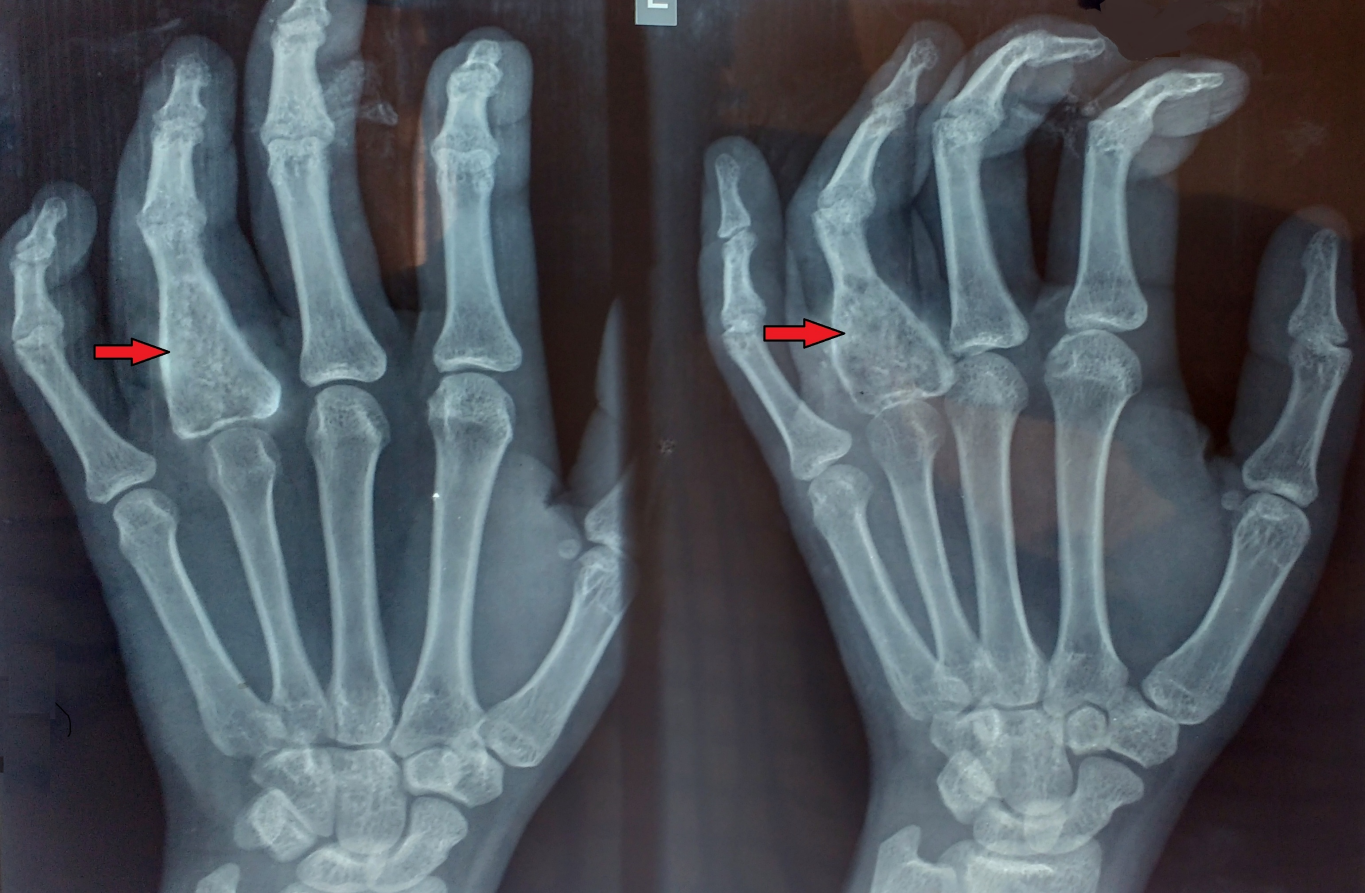
Follow-up radiograph at two years Shows good incorporation of the bone graft and no signs of recurrence

Postoperatively, active and passive range of movement exercises of the digits and wrist were begun. After gaining functional range of movement at MCP and PIP joints, gradual hand strengthening was initiated. The patient was instructed to refrain from contact sports and lifting heavy objects for three months. During further follow-ups his grip strength was normal with good functional recovery. At two years follow-up there was complete healing of the lesion with no evidence of recurrence (Figure 4).

## Discussion

Giant cell tumour of the bone is benign and locally aggressive with an uncertain biological behaviour. Most giant cell tumours occur in patients between 20 to 40 years of age [1]. The most common location for GCT is adjacent to the knee joint, located in the epiphysis [1]. Of all reported GCTs only two percent are found in the hand and seem to be different from conventional GCT as recurrence is more rapid in the hand than in other locations [2].

Pain and swelling are usually the first symptoms of GCT, especially when it occurs over the extremities like the hand due to its proximity to the surface. The pain is usually insidious at onset with progressive worsening of symptoms. In a few cases, the tumour may grow quickly, with thinning and destruction of the bone cortex and invasion of the adjacent soft tissues. However, a pathological fracture at the lesion may result in acute pain [3-4].

Giant cell tumour has typical radiographic features such as an epiphyseal eccentric location, expansile and lytic lesion with thinning of the cortex. However, there is absence of internal calcifications, marginal sclerosis, or periosteal reaction [4]. Computed tomography (CT) helps to confirm the absence of calcified matrix, delineates the margins of the lesion, and depicts the extent of subcortical bone loss. Magnetic resonance imaging (MRI) is considered the best method for deciding the extent of the tumour, particularly any cortical breach and soft tissue extension, if present [5]. It is of utmost importance in demonstrating recurrence of the tumour [3].

Enneking first described the staging of GCT of the bone, subsequently followed by Campanacci who gave the radiological classification. Three stages of the local tumour which correlate to the aggressiveness and risk of local recurrence are depicted. Campanacci's Grade 1 is a cystic lesion (latent) with sclerosed margins. Grade 2 (active) is the most common type where the cortex is thin but there is no extension into the surrounding soft tissue. In Grade 3, the cortex is perforated and the tumour is large in size, destroying the cortex and invading the surrounding tissues. The highest rate of recurrence is observed in tumours that are in Grade 3 [4-5].

Grossly, GCT of the bone is brown in colour, solid, with areas of necrosis and haemorrhage. Histologically, the neoplasic tissue shows highly vascularised stroma interspersed with oval or fusiform cells, with multinucleated giant cells of the osteoclast type [6]. Mitotic figures may be present, but without abnormalities [5].

Differential diagnosis includes benign lesions like aneurysmal bone cyst, brown tumour of hyperparathyroidism, giant cell reparative granuloma, early stages of metastatic disease, and multiple myeloma. Radiologically, they may appear similar to GCT, but it is the histological examination that can differentiate these tumours [4-5].

Primary modality of treatment of GCT is surgical [3, 6]. The surgical procedure should be thoroughly planned and individualized as the tumour has varied response to different modalities with the greatest challenge being able to avoid local recurrence [7-8]. Compared with GCT in more common locations, tumours of the hand are presented in later stages with greater bone destruction, which complicates the treatment [5, 7]. The types of treatment described in the literature are: curettage, curettage with bone graft, amputation, resection with reconstruction, and radiotherapy [6]. Curettage, simple or with an adjuvant like phenol or cryotherapy, whether in isolation or associated with bone graft, is the most common form of treatment, but its rate of recurrence reaches around 20% to 90% [2, 7]. In cases of recurrence, a second similar local intralesional procedure is typically sufficient in cases that are detected early [9]. Daniel, et al. reported GCT of the middle phalanx treated with curettage and bone grafting, which at nine months recurred and was successfully treated by excision of lesion and allograft replacement. Wittig, et al. reported three cases of phalangeal GCT managed with curettage, cryosurgery, and cementation. Out of three cases of GCT of the hand reported by Patel, et al. treated with curettage and bone grafting, two had local recurrence and were managed with ray resection [2].

Amputation, although it reduces the recurrence rate, is cosmetically disfiguring and causes loss of functionality of the limb. Resection with reconstruction of the base of the proximal phalanx for articulation with the metacarpus can be done with a bone graft, polymethylmethacrylate (PMMA) cement or prosthesis, which reconstructs the functional and structural integrity of the metacarpophalangeal joint [6].

Medical therapy and radiotherapy can alter the management of GCT of bone, especially in multifocal and metastatic disease and in cases of local recurrences. As radiotherapy is associated with malignant transformation, it should also not be used as the primary procedure. Medical therapies like bisphosphonates and denosumab, as an adjuvant therapy for giant cell tumour of bone, have demonstrated a lower local recurrence rate with more promising results in stage III diseases [10].

In the present case, extended curettage with phenol application and bone grafting procedure was carried out in accordance with clinical condition and radiological findings. The patient had regular follow-up for two years during which no signs of recurrence were seen and the patient had complete functional recovery.

With the appropriate surgical technique, the rate of recurrence varies from five percent to 10% [1, 5] and the majority occurs around 12 to 18 months after therapy [7]. In the hands, the recurrence rate is higher [7]. A literature review indicates that patients with recurrence should be carefully followed up due to the greater malignant potential of the recurrent disease as well as metastasis and the higher propensity to metastasis in the lungs as compared to primary tumour [4-5].

## Conclusions

GCT in the hand is a rare, benign, locally aggressive tumour. It evolves earlier than GCT in other locations. The diagnosis is based on the clinical, radiological, and histopathological findings with primary treatment being surgical. Each case should be assessed individually to ensure adequate treatment, aiming to prevent recurrences and functional limitations. In view of the comparative rarity of a tumour arising from the phalanges of the ﬁnger and the higher rate of recurrence after curettage, the contrary outcome observed in this case makes it worth reporting.
